# DCIS knowledge of women choosing between active surveillance and surgery for low-risk DCIS

**DOI:** 10.1016/j.breast.2024.103764

**Published:** 2024-07-02

**Authors:** E.G. Engelhardt, R.S.J.M. Schmitz, M.A. Gerritsma, C.M.T. Sondermeijer, E. Verschuur, J.H.E. Houtzager, R. Griffioen, N. Bijker, R.M. Mann, V. Retèl, F.H. van Duijnhoven, J. Wesseling, E.M.A. Bleiker, Grand Challenge PRECISION Consortium, Alastair Thompson, Serena Nik-Zainal, Elinor J. Sawyer, Helen Davies, Andrew Futreal, Nicholas Navin, E. Shelley Hwang, Jos Jonkers, Jacco van Rheenen, Fariba Behbod, Esther H. Lips, Marjanka Schmidt, Lodewyk F.A. Wessels, Daniel Rea, Proteeti Bhattacharjee, Hilary Stobart, Deborah Collyar, Donna Pinto, Marja van Oirsouw, S. Alaeikhanehshir, L. Elshof

**Affiliations:** m Baylor College of Medicine, Houston, TX, USA; n University of Cambridge, Cambridge, UK; o King's College London, London, UK; p Anderson Cancer Center, Houston, USA; q Duke University School of Medicine, Cancers 2022, 14, 3259 10 of 13 Durham, NC, USA; r Netherlands Cancer Institute, Amsterdam, the Netherlands; s Kansas University Medical Center, Kansas, USA; t University of Birmingham, Birmingham, UK; u Independent Cancer Patients' Voice, UK; v Patient Advocates in Research, USA; w dcis411, USA; x Borstkanker Vereniging Nederland, the Netherlands; a Division of Psychosocial Research and Epidemiology, Netherlands Cancer Institute, Plesmanlaan 121, 1066 CX, Amsterdam, the Netherlands; b Division of Molecular Pathology, Netherlands Cancer Institute, Plesmanlaan 121, 1066 CX, Amsterdam, the Netherlands; c Biometrics Department, Netherlands Cancer Institute, Plesmanlaan 121, 1066 CX, Amsterdam, the Netherlands; d Borstkanker Vereniging Nederland (breast cancer patient association), Domus Medica, Marecatorlaan 1200, 3528 BL Utrecht, the Netherlands; e Department of Radiation Oncology, Amsterdam University Medical Center, Amsterdam UMC, locatie AMC, Meibergdreef 9, 1105 AZ Amsterdam Zuidoost, Amsterdam, the Netherlands; f Department of Radiology, Netherlands Cancer Institute, Plesmanlaan 121, 1066 CX, Amsterdam, the Netherlands; g Department of Radiology, Radboud University Medical Center, Geert Grooteplein Zuid 10, 6525 GA Nijmegen, the Netherlands; h Department of Surgery, Netherlands Cancer Institute, Plesmanlaan 121, 1066 CX, Amsterdam, the Netherlands; i Department of Pathology, Netherlands Cancer Institute, Plesmanlaan 121, 1066 CX, Amsterdam, the Netherlands; j Department of Pathology, Leiden University Medical Center, Albinusdreef 2, 2333 ZA Leiden, the Netherlands; k Department of Clinical Genetics, Leiden University Medical Center, Albinusdreef 2, 2333 ZA Leiden, the Netherlands; l Department op Clinical Genetics, Netherlands Cancer Institute, Plesmanlaan 121, 1066 CX, Amsterdam, the Netherlands

**Keywords:** Ductal carcinoma in situ, Knowledge, Information provision, Shared decision making

## Abstract

**Background:**

Ductal carcinoma in situ (DCIS) can progress to invasive breast cancer (IBC), but often never will. As we cannot predict accurately which DCIS-lesions will or will not progress to IBC, almost all women with DCIS undergo breast-conserving surgery supplemented with radiotherapy, or even mastectomy. In some countries, endocrine treatment is prescribed as well. This implies many women with non-progressive DCIS undergo overtreatment. To reduce this, the LORD patient preference trial (LORD-PPT) tests whether mammographic active surveillance (AS) is safe by giving women with low-risk DCIS a choice between treatment and AS. For this, sufficient knowledge about DCIS is crucial. Therefore, we assessed women's DCIS knowledge in association with socio-demographic and clinical characteristics.

**Methods:**

LORD-PPT participants (N = 376) completed a questionnaire assessing socio-demographic and clinical characteristics, risk perception, treatment choice and DCIS knowledge after being informed about their diagnosis and treatment options.

**Results:**

66 % of participants had poor knowledge (i.e., answered ≤3 out of 7 knowledge items correctly). Most incorrect answers involved overestimating the safety of AS and misunderstanding of DCIS prognostic risks. Overall, women with higher DCIS knowledge score perceived their risk of developing IBC as being somewhat higher than women with poorer knowledge (p = 0.049). Women with better DCIS knowledge more often chose surgery whilst most women with poorer knowledge chose active surveillance (p = 0.049).

**Discussion:**

Our findings show that there is room for improvement of information provision to patients. Decision support tools for patients and clinicians could help to stimulate effective shared decision-making about DCIS management.

## Highlights

• Low-risk DCIS is surgically treated, but not all will progress to breast cancer.
• LORD trial: women can opt for conventional treatment or for active surveillance.
• Adequate knowledge about DCIS is essential for patients to make an informed choice.
• Overall DCIS knowledge about safety of active surveillance was poor.
• DCIS knowledge level is associated with risk perception and treatment choice.

## 1 Introduction

Ductal carcinoma in situ (DCIS) is a potential precursor to invasive breast cancer and accounts for approximately 13 % of newly diagnosed breast (pre)malignancies in the Netherlands [1]. Current clinical treatment guidelines recommend surgery, i.e., breast-conserving surgery, almost always supplemented with radiotherapy, or even mastectomy [2]. In some countries, additional endocrine treatment is also recommended. However, available evidence suggests that as much as 80 % of DCIS cases could be indolent (i.e., the lesion may never progress to invasive breast cancer) [[3], [4], [5], [6], [7]]. To reduce overtreatment of women diagnosed with low-risk DCIS, the LORD patient preference trial (LORD-PPT) [8] is investigating whether active surveillance is a safe alternative to conventional treatment (i.e., surgery with/without radiotherapy). Women participating in the LORD-PPT are given a choice between active surveillance and conventional treatment. However, as there is no definitive evidence proving the safety of active surveillance for DCIS compared to conventional treatment yet, this can be a complex decision fraught with uncertainty for both patients and clinicians. Patients must process complex medical information shortly after having received the diagnosis and make a subjective trade-off between the pros and cons of conventional treatment vs. active surveillance. On the one hand, if women forego treatment, they avoid the harms caused by surgery and radiotherapy. On the other hand, not undergoing treatment can cause anxiety and worry about the DCIS progressing to invasive breast cancer. This can also negatively impact women's quality of life. Which option to choose depends on patients' informed preferences. For women to be able to effectively participate in decision making about DCIS management, good knowledge about DCIS and the pros and cons of surveillance and surgery with or without radiotherapy is needed.

Healthcare professionals play a key role in information provision. It has been reported that healthcare professionals find it challenging to explain to their patients, what DCIS is, its difference with invasive breast cancer, and its potential prognostic implications [9]. Additional challenges are the use of consistent terminology to describe DCIS. Inconsistencies can cause confusion among patients, especially those with low health literacy. It has repeatedly been reported in the literature that DCIS knowledge among women diagnosed with DCIS is poor [[10], [11], [12], [13], [14], [15], [16]]. Misconceptions about what DCIS is and its potential prognostic consequences are prevalent and associated with women experiencing more confusion and worry about the diagnosis and treatment both in the short- and long-term [[10], [11], [12], [13], [14], [15], [16]]. Furthermore, many women treated for DCIS tend to overestimate their risk of (distant) recurrence [[11], [12], [13], [14],^17^]. Women who overestimated their risks more often reported frequent worry and lower mental health [17]. Disparities in DCIS knowledge were identified by education level, socioeconomic status, ethnicity, and literacy [9]. Previous studies have shown that even among women who undergo surgery (and other adjuvant treatments), worry about DCIS recurrence or progression was one of the key factors that affect women's quality of life, with generally about a third of women reporting that they experience moderate to high levels of anxiety or fear of recurrence [9,18,19]. A complicating factor in decision-making for women with low-risk DCIS is that there is uncertainty and divergent estimates of their risk of developing invasive breast cancer.

In the context of the LORD-trial, women with low-risk DCIS are making a choice that can have significant impact on their quality of life. Women's participation in decision-making, particularly in the weighing of the pros and cons of conventional treatment vs. active surveillance, is key to choosing the option that best suits their individual situation. It is important that the choice these women make, is based on adequate understanding of what DCIS is and the potential consequences in the short- and the long term of undergoing vs. foregoing treatment. Insights into DCIS knowledge of women currently participating in the trial, can help us identify knowledge gaps, opportunities to improve doctor-patient communication, and unmet decision support needs. Therefore, in the current study, we: 1) evaluated DCIS knowledge among women participating in the LORD-PPT and 2) assessed whether patient- and disease-related factors are associated with women's level of DCIS knowledge.

## 2 Methods

### 2.1 Study population

The current study is embedded within the ongoing LORD-PPT for which women are being recruited in 56 hospitals across the Netherlands. Briefly, women can be included if: ≥45 years with an ASA 1–2 score, diagnosed with unilateral DCIS without invasive component that is grade 1 or 2, any size, ER-positive and HER2-negative detected through screening. Women with symptomatic DCIS, a history of (breast) malignancy or DCIS, and women (or family members) with a proven mutation increasing the risk of breast cancer are excluded. Women eligible to participate in the LORD trial are informed about their diagnosis and the DCIS management options – including active surveillance if they choose to participate in the LORD – by a breast surgeon and/or a nurse practitioner/nurse specialized in breast cancer. Clinicians participating in the LORD trial are not prescribed what to say to patients, they inform patients about the diagnosis and treatment options as they would do as part of care as usual. If the patient is interested to participate, they receive written patient information about the LORD trial and generally also a pamphlet with frequently asked questions about DCIS and the trial designed by the patient advocates involved in the study. Patients may also receive any additional informational brochures routinely provided to patients at that specific hospital. Women eligible for the LORD-trial who agree to participate and had completed the baseline questionnaire by June 17, 2022 were selected for the current study. The LORD-trial was approved by the medical research ethics committee of the Netherlands Cancer Institute (NL55612.031.16).

### 2.2 Procedures and measures

Information regarding patient characteristics, DCIS knowledge, and DCIS management strategy choice were collected with the baseline study questionnaire. Table 1 provides an overview and description of the variables used in the current study. Patients received the baseline questionnaire immediately after the consultation with their breast surgeon and/or nurse practitioner/nurse specialized in breast cancer in which the diagnosis and DCIS management strategies had been discussed. Participants were instructed to complete the questionnaire within one week of this consultation. Of the women participating in the current study, 62 % returned their questionnaires within 2 weeks. The average return rate for the baseline questionnaire of women participating in the larger LORD trial is approximately 89 %. Clinical data were collected by trained data managers from patients' electronic health records. For this study, information on DCIS grade and DCIS size was extracted from the LORD-trial's electronic data capture system (see Table 1 for description).

**Table 1:** Overview of measures.

	Description	Operationalization for the analyses
Outcome measure		
DCIS knowledge	Knowledge was measured using seven statements based on the DCIS knowledge originally developed by Bluman et al. [15] and extended/revised by Parikh et al. [16]. Patients were asked to indicate whether each statement is true or false. 1 . During a physical examination, the physician can always feel the DCIS lesion. (F) 2 . DCIS can spread to other parts of the body over time. (F) 3 . If left untreated, DCIS can progress into invasive breast cancer. (T) 4 . Women who have had DCIS are more likely to develop invasive breast cancer in the future than women who have never had DCIS. (T) 5 . DCIS can always be detected on a mammogram. (F) 6 . Women who have had DCIS are more likely to develop breast cancer in the other breast than women who have never had DCIS. (T) 7 . If a woman with DCIS undergoes annual mammography, any growth of DCIS will always be detected before it can cause harm. (F) T = True; F= False	Each correct answer was rated with one point. If at least one knowledge item had been answered, other item(s) left blank were considered incorrect answers in the analyses (proportion non-response on knowledge items: 12 people did not answer questions 2–4, 15 people did not answer question 5, 11 people did not answer question 6, and 20 people did not answer question 7). The total number correct answers were calculated (min = 0 and max = 7). We next dichotomized the score into: - Low knowledge score = three or fewer questions were answered correctly - High knowledge score = four or more questions were answered correctly The cut-off is self-selected as no validated cut-off exists and reflects the proportion of patients who are able to correctly answer more than half of the seven questions.
Potential predictors		
Perception of risk of developing invasive breast cancer	Perception of breast cancer risk compared to general Dutch population (categorical assessment) is based on Lerman et al. [21] Perception of the risk of developing invasive breast cancer was measured using a multiple-choice question: *Compared with the average woman of your age from the Dutch population, you think your risk of developing invasive breast cancer is …*	Respondents could select one of the following answering categories lower: the risk is: - Lower - Equal - Slightly elevated - Moderately elevated - Highly elevated There is uncertainty in the scientific literature regarding the risk of developing invasive breast cancer after DCIS. Taking the width of estimates into account, ‘slightly to moderately elevated’ risk is considered accurate perception of risk.
Socio-demographic and psychological characteristics	Age Respondents reported their age at the time they completed the questionnaire.	Reported ages were categorized as follows: −45–54 years −55–64 years −65 years and older
	Education level Educational level was measured using a multiple-choice question: *What is your highest completed educational level?* Respondents were provided with a list of degrees as well as an open text box if none of the options provided were applicable.	Reported educational level was categorized as follows: - Low = elementary school, secondary vocational education - Moderate = high school, post-secondary vocational education - High = higher vocational education or university
	Tolerance of uncertainty Tolerance of uncertainty was measured using the Uncertainty Intolerance Scale (IUS) [22], which consists of 12 items, scored on a 5-point Likert scale from completely disagree to completely agree.	A total score was calculated by adding the scores for all the items together (minimum = 12 and maximum = 60). A data-driven cut off was used in this study as there is no official cut-off for the IUS. Here we defined high vs. low intolerance of uncertainty as follows: - High tolerance = sum scores ≥36 (i.e., 75 % of the maximum achievable score) - Low tolerance = sum scores <36
	Anxiety and depressions level Level of anxiety and depression at diagnosis was measured using the Hospital Anxiety and Depression Scale (HADS) [23]. The HADS consists of an anxiety (7 items) and depression (7 items) subscale. Here we report the score of each subscale separately.	According to the official cut offs [23] an anxiety or depression score of seven or lower is considered “No clinical anxiety/depression disorder”, eight to ten is considered “possible/mild clinical anxiety/depression disorder” and >10 is considered “clinical anxiety/depression disorder”. We categorized the sum score as follows: - Not elevated = sum score <10 - Elevated = sum score ≥11 The listed cut-offs apply to both the anxiety and depression scales.
Clinical characteristics	DCIS grade DCIS grade was defined as grade one or grade two following the WHO classification of breast tumors [24].	Categorized as follows: - Grade 1 (low grade) - Grade 2 (intermediate grade)
	DCIS lesion size Size was defined as the largest diameter of the span of suspicious calcifications on mammography.	Categorized as follows: - Smaller than 20 mm −20–49 mm −50 mm or larger
Chosen DCIS management strategy	Question used: *Which management option have you chosen?* Respondents were provided with multiple-choice answering options. Also, as the questionnaire was completed directly after the consultation with the surgeon, some patients might not have made a decision yet. They also had the option to indicate this and the option they were leaning towards. This information was used to categorize chosen management strategy if no final decision was available at date of data extraction (was necessary for <10 women).	Categorized as follows: - Conventional treatment = mastectomy or breast conserving surgery with radiotherapy or breast conserving surgery without radiotherapy - Active surveillance = no surgery, active surveillance for 10 years
Trust in oncologist	Trust in oncologist was measured using the five-item Trust in Oncologist Scale (short form) by Hillen et al. [25] scored on a 5-point Likert scale from completely disagree to completely agree.	The score is calculated by adding the score for the five items together and dividing that by five. This provides a final rating between one and five, with higher scores reflecting greater trust. - Low level of trust = scores smaller than 3 - Neutral = scores between 3 and 4 - High level of trust = scores greater than 4

### 2.3 Statistics

Descriptive statistics were used to describe the study population. In total, eight respondents did not answer any knowledge items and were excluded from all analyses related to DCIS knowledge. Chi square, Fisher's exact and Mann-Whitney U tests were used as appropriate to investigate potential associations between patients' level of DCIS knowledge (dichotomized as: low vs. high level of DCIS knowledge) and patient and disease characteristics (see Table 1 for overview of potential predictors) and treatment choice (i.e., conservative treatment or active surveillance). Two-sided P-values ≤0.05 are considered statistically significant. The p-values were adjusted for multiple comparisons using the Benjamini-Hochberg procedure to control the false discovery rate [20]. All analyses were performed using IBM SPSS version 27.

## 3 Results

In total, questionnaire data was available of 376 women with low-risk DCIS participating in the LORD-trial who had completed the DCIS knowledge items. Median age was 59 years (range: 45–83) and educational level was evenly distributed (low: 35 %, moderate: 30 %, and high: 35 %) (Table 2). Of participants for whom clinical data had been collected at the time of data extraction, 46 % had grade 1 DCIS and 77 % had small lesions (i.e., <20 mm). In total, 77 % chose active surveillance as management strategy for their DCIS. Most participants (74 %) had a high tolerance for uncertainty and 87 % had a high level of trust in their surgical oncologist. Almost all participants reported that decision-making about DCIS management strategy had been patient-driven (72 %) or shared (26 %).

**Table 2:** Association between DCIS knowledge and patient and disease characteristics (n_col_ (%)).

	Participants N = 376	Low knowledge score n = 249	High knowledge score n = 127	P^b^
Age				
*45-54 years*	132 (35)	79 (32)	53 (42)	0.293
*55-64 years*	124 (33)	86 (35)	38 (30)	
*65 years and older*	120 (32)	84 (34)	36 (28)	
Education level				
*Low*	132 (35)	88 (35)	44 (35)	0.605
*Moderate*	111 (30)	78 (31)	33 (26)	
*High*	133 (35)	83 (33)	50 (39)	
DCIS grade^a^				
*Grade 1*	124 (46)	84 (46)	40 (44)	0.800
*Grade 2*	147 (54)	97 (54)	50 (56)	
*Missing*	105	68	37	
DCIS lesion size^a^				
*Smaller than* 20* *mm	191 (77)	133 (80)	58 (72)	0.409
*20-*49* *mm	44 (18)	25 (15)	19 (24)	
50* *mm *or larger*	13 (5)	9 (5)	4 (5)	
*Missing*	128	82	46	
Chosen DCIS management strategy				
*Conventional treatment*	84 (23)	46 (19)	38 (31)	0.049
*Active surveillance*	286 (77)	201 (81)	85 (69)	
*Unknown*	6	2	4	
Tolerance of uncertainty				
*Low tolerance*	97 (26)	62 (25)	35 (28)	0.605
*High tolerance*	278 (74)	187 (75)	91 (72)	
*Missing*	1	0	1	
HADS Anxiety level				
*Not elevated*	317 (84)	215 (86)	102 (80)	0.286
*Elevated*	59 (16)	34 (14)	25 (20)	
HADS Depression level				
*Not elevated*	337 (90)	229 (92)	108 (85)	0.183
*Elevated*	39 (10)	20 (8)	19 (15)	

DCIS = ductal carcinoma in situ; HADS = hospital anxiety and depression scale; IQR = interquartile range.

N_Col_ = column percent and shows the proportion of observations in each row from among those in the column.

Percentages do not always add up to 100 % due to rounding.

a High proportion of missing as data collection from electronic patient records had not yet been completed at the time of data extraction.

b P-value based on Chi-square, Fisher's exact or Mann-Whitney test as appropriate; p-value ≤0.05 considered significant. The p-values were adjusted for multiple comparisons using the Benjamini-Hochberg procedure to control the false discovery rate.

### 3.1 RQ 1 level of DCIS knowledge

**Fig. 1:**
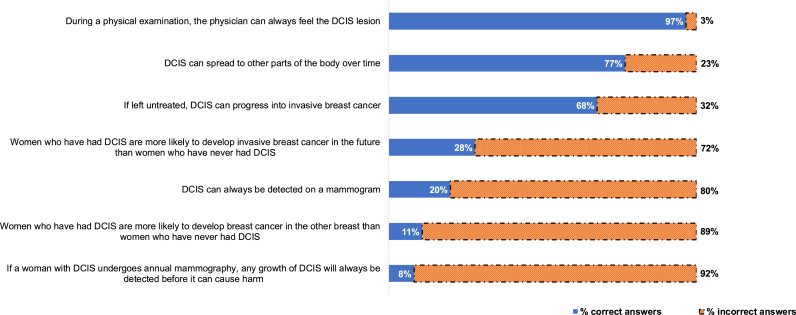
Overall percentage (in)correct answers on the DCIS knowledge items (N = 376) DCIS = ductal carcinoma in situ.

**Fig. 2:**
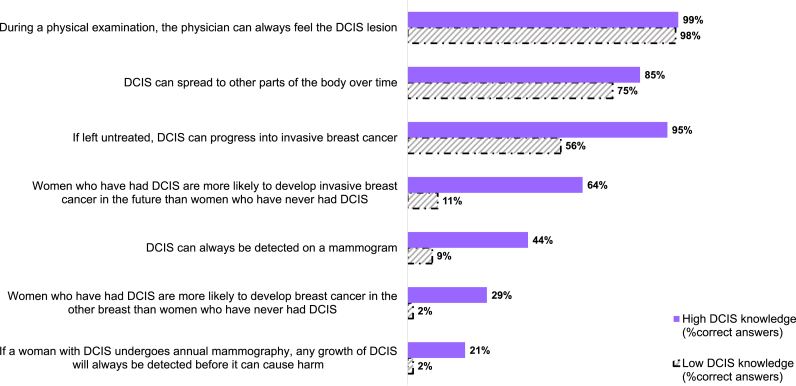
Percentage (in)correct answers on the DCIS knowledge items by level of DCIS knowledge (N = 376) DCIS = ductal carcinoma in situ.

Overall, 249 out of 376 (66 %) participants had a low knowledge score (i.e., three or fewer items out of seven correctly answered). Fig. 1 provides an overview of the overall percentage of (in)correct answers for the individual items. The statement with the highest proportion of incorrect answers was: 1) *If a woman with DCIS undergoes annual mammography, any growth of DCIS will always be detected before it can cause harm* (92 % incorrect answers). The knowledge statement with the highest proportion of correct answers was: “*During a physical examination, the physician can always feel the DCIS lesion*” (false) (97 % was correct). Fig. 2 provides an overview of the proportion of correctly answered knowledge statements by whether women have low vs. high DCIS knowledge. Women with a low DCIS knowledge score performed significantly worse on all knowledge statements with the exception of “*During a physical examination, the physician can always feel the DCIS lesion”*. Most women in the high knowledge group poorly performed on the statements: “*Women who have had DCIS are more likely to develop breast cancer in the other breast than women who have never had DCIS*” and “*If a woman with DCIS undergoes annual mammography, any growth of DCIS will always be detected before it can cause harm*”. Finally, when participants were asked to indicate whether their risk of developing invasive breast cancer was lower, equal, or slightly, moderately or highly elevated compared to the general Dutch population, most participants indicated that they had either a lower/equal (43 %) or slightly/moderately elevated (57 %) risk.

### 3.2 RQ 2 association between patient and disease related factors and DCIS knowledge

We found two statistically significant associations. First, participants’ perception of their own breast cancer risk was associated with DCIS knowledge level (p-value: 0.049) (Table 2). More women with low DCIS knowledge compared to those with high DCIS knowledge perceived their risk of developing invasive breast cancer to be lower/equal than the general Dutch population (49 % vs. 31 %). Second, participants with low DCIS knowledge (81 %) more often chose active surveillance compared to the participants with high DCIS knowledge (69 %) (p-value: 0.049). No statistically significant association was found between with DCIS knowledge and age, education level, trust in oncologist, tumor characteristics or psychosocial characteristics.

## 4 Discussion

In the current study, we assessed DCIS knowledge among women diagnosed with low-risk DCIS participating in the LORD-PPT. These women made a choice between conventional treatment (i.e., surgery with/without radiotherapy) and active surveillance (yearly mammogram only) for DCIS. Overall DCIS knowledge was low for two-thirds of participants. Most women gave incorrect answers to questions relating to the safety of active surveillance and on the potential impact of a DCIS diagnosis on the probability of experiencing invasive breast cancer in the future. Higher perceived breast cancer risk was associated with better DCIS knowledge. In addition, women with better DCIS knowledge more often chose conventional treatment. Education level, age at diagnosis, trust in oncologist, tolerance for uncertainty and all clinical characteristics considered were not associated with DCIS knowledge in our sample.

The high proportion of participants with low scores on knowledge in our study population is in line with previous studies assessing DCIS knowledge [15,16]. The observation that DCIS knowledge is associated with choice of DCIS management strategy and that more women in the low knowledge group chose to forego treatment raises the question whether women had made informed choices? If they had not, is that concerning? From an ethical perspective it is always important that patients are adequately informed to make treatment decisions. Participants in our study population perceived high decisional control, i.e., shared or patient-driven decision making. If such decisions are made without sufficient understanding of the disease and the treatment options, patients might be more likely to experience decisional regret and distress when unexpected negative outcomes occur (e.g., progression of the DCIS lesion). We do not yet have sufficient follow-up for the women in our sample to assess the impact of decision-making on these outcomes. However, the literature suggests that these are valid concerns.

Our findings also highlight a need for a patient decision aid alongside information provision by healthcare professionals to help patients become better informed on such a complex topic. Studies assessing women's experiences have consistently identified knowledge gaps and misperceptions as a source of distress and elevated worry/anxiety among women treated for DCIS [[15], [16], [17], [18], [19],^26^]. Rosenberg et al. reported based on data from a large cohort of US-based women that in general, a DCIS diagnosis was perceived as confusing and distressing and that treatment decisions regularly seemed to be made despite patients having a limited/incomplete understanding of the disease, its risks, and the pros and cons of different treatment options [10]. Women reported experiencing significant uncertainty associated with knowledge gaps as well as persistent decisional regret even a long time after they had undergone treatment for DCIS [10]. For women with low-risk DCIS as well as the healthcare professionals treating them, there currently is much uncertainty. Yet, in spite of all the unknowns, treatment decisions must be made. Communicating the available information to patients effectively could help them make better informed decisions, cope better with the uncertainty, and limit decisional regret.

We found an association between DCIS knowledge and perception of the risk of developing invasive breast cancer compared to the general Dutch population. Particularly the women in the low knowledge level group seemed to have a strong trust in the safety of the active surveillance approach. We also observed that the women in the low DCIS knowledge group (81 %) significantly more often chose active surveillance compared to the women in the high knowledge group (69 %). These findings suggest that greater awareness of the uncertainties associated with DCIS and the treatment options might influence women's risk perception and by extension choice of DCIS management strategy. We do not have the data to disentangle these observed relationships further. However, our findings suggest that it is important to pay attention to information provision about potential risks associated with a DCIS diagnosis and the different options women have to manage their DCIS. Efforts are ongoing to improve estimation of the outcomes relevant for women with DCIS to allow healthcare professionals to continue to improve information provision to patients.

It is important to note that the knowledge scores we observed in this study need not reflect the quality of information provided by healthcare professionals or in the LORD trial but do highlight that there is room for improvement. Many factors could have influenced what knowledge women retained and to what extent they understood the complexities of DCIS and the available treatment options. Interestingly, having a higher educational level was not associated with higher knowledge scores in our sample. Perhaps comprehension of the information provided is not the only or most crucial aspect in patients' decision making. Women's perception of their healthcare provider's preference for management strategy might play a key role in their choice. Thus, if there is a perceived preferred option by an expert (i.e., their doctor) there might be less impetus to actively listen to all the complex medical information. In such instances patients might not have processed as much of the information they received as they would have if they had to weigh the options without a perceived recommendation by their doctor. Unfortunately, consultations were not observed or recorded, thus, we do not have insights into their content, or the quality of the information provided. To our knowledge there are no studies evaluating the content of the consultation on DCIS treatment we can draw on. But studies have shown that unintended steering during doctor-patient consultations is common [[27], [28], [29]]. There is a need to carry out studies to assess the quality of information provision during doctor-patient consultations on DCIS to be able to identify areas requiring improvement so that we can provide support more effectively to both healthcare professionals and patients.

Our study provides important insights into the level of DCIS knowledge among women with low-risk DCIS who were given a choice between conventional treatment and active surveillance in the context of a patient preference trial. Key strengths of our study are the large sample size, and that DCIS knowledge was measured within a short time after the consultation in which patients received information about their DCIS diagnosis and treatment options. Our study also has some limitations. Although we used a DCIS knowledge questionnaire that is based on a questionnaire that had been used in previous studies [15,16], the way in which some of the knowledge items are formulated could have caused problems with interpretation leading to incorrect answers, particularly the use of the term “always” in the statements. For example, the item “*If a woman with DCIS undergoes annual mammography, any growth of DCIS will always be detected before it can cause harm*”; if respondents overlooked the term “always” this could lead to an incorrect interpretation of the statement. Yet, it is important to note that in our data women who incorrectly answered the item listed above, tended to also answer other items zooming in on potential risks of active surveillance incorrectly and in the same direction. Also, defining what is essential DCIS knowledge is subjective. Arguably, other relevant knowledge components might have been missed. Further, although all patients should have received the flyers with frequently asked questions about DCIS, we cannot be sure all participants received it. Finally, we used a categorical variable to assess risk perception. This measurement might be a little less sensitive than asking participants to provide a numerical risk estimate.

In conclusion, our findings underscore that there is room for improvement regarding information provision. Decision support tools (e.g., a patient decision aid and communication guides for clinicians) can help healthcare professionals facilitate informed decision-making about DCIS management [30]. For the Dutch context there is not yet a decision aid for women with DCIS [31]. This need is particularly urgent in the Dutch context as the high accrual into the LORD-PPT and participants’ marked preference for active surveillance suggests that if proven safe, active surveillance is likely to become part of the standard of care for women with low-risk DCIS.

## Funding

This work was supported by 10.13039/501100000289Cancer Research United Kingdom and by KWF Dutch Cancer Society (ref. C38317/A24043). Research at the Netherlands Cancer Institute is supported by institutional grants of the Dutch Cancer Society and of the 10.13039/501100002999Dutch Ministry of Health, Welfare and Sport, The Netherlands. The funders had no role in the study design, data collection, analysis and interpretation of data; the writing of the manuscript; or the decision to submit the manuscript for publication.

## CRediT authorship contribution statement

**E.G. Engelhardt:** Writing – review & editing, Writing – original draft, Formal analysis, Data curation, Conceptualization. **R.S.J.M. Schmitz:** Writing – review & editing, Data curation, Conceptualization. **M.A. Gerritsma:** Writing – review & editing, Project administration, Data curation. **C.M.T. Sondermeijer:** Writing – review & editing, Project administration, Data curation. **E. Verschuur:** Writing – review & editing. **J.H.E. Houtzager:** Writing – review & editing, Data curation. **R. Griffioen:** Writing – review & editing, Data curation. **N. Bijker:** Writing – review & editing. **R.M. Mann:** Writing – review & editing. **V. Retèl:** Writing – review & editing. **F.H. van Duijnhoven:** Writing – review & editing. **J. Wesseling:** Writing – review & editing, Supervision, Funding acquisition. **E.M.A. Bleiker:** Writing – review & editing, Supervision, Conceptualization.
